# Magnetically Controlled Two-Dimensional Charge Transport in Repulsive Nanostructured Potentials

**DOI:** 10.3390/nano16110661

**Published:** 2026-05-24

**Authors:** Orion Ciftja, Cleo L. Bentley

**Affiliations:** Department of Physics, Prairie View A&M University, Prairie View, TX 77446, USA; clbentley@pvamu.edu

**Keywords:** charge transport, nanostructured potential, magnetic confinement, cyclotron motion, nanomaterials and devices

## Abstract

We study the planar dynamics of a charged particle subjected to a radially repulsive inverted harmonic potential and a perpendicular uniform magnetic field, a configuration that is relevant to nanoscale-charged transport and confinement in low-dimensional systems. The competition between the destabilizing central repulsion and magnetic field-induced rotational motion gives rise to rich trajectory behavior, including spiraling, unbounded escape, and parameter-dependent quasi-confined motion. The governing coupled differential equations of motion are solved analytically. The resulting trajectories are classified as functions of system parameters. The proposed framework provides insight into charge carrier dynamics in nanostructured environments such as quantum wells, 2D materials, and plasma-like nanosystems, where effective repulsive potentials may arise from external gating or collective interactions. In addition, the model offers a classical analogue for interpreting features associated with magnetic confinement in non-equilibrium or unstable regimes. These results contribute to the theoretical foundation for designing and controlling charged particle motion in emerging nanomaterials and devices.

## 1. Introduction

The dynamics of charged particles in electromagnetic fields underpin a wide range of phenomena in both classical and quantum systems, with direct relevance to nanoscale transport in magnetic materials, two-dimensional (2D) platforms, and plasma-like environments [1,2,3,4,5,6,7]. In particular, understanding charge motion under combined electric and magnetic fields is essential for the design of functional nanomaterials and devices. These materials include a diverse number of magnetically controlled classically behaving structures as well as various quantum counterparts that behave as quantum Hall systems [8,9,10,11,12,13,14,15,16,17,18,19,20]. In this work, we investigate the planar motion of a charged particle in a 2D setting subjected to a central repulsive potential and a uniform magnetic field applied perpendicular to the plane. This minimal model captures the interplay between destabilizing electrostatic effects and magnetic confinement, providing a useful framework for describing charge carrier dynamics in low-dimensional nanostructures where effective repulsive interactions and external fields can be engineered.

In classical electrodynamics, the motion of charged particles is governed by the Lorentz force, which dictates their response to external electric and magnetic fields [21,22,23,24,25,26,27]. In a 2D geometry with a perpendicular uniform magnetic field, particles undergo cyclotron motion [28], forming circular trajectories with a frequency set by the charge-to-mass ratio and field strength. This fundamental behavior underlies a variety of nanoscale applications, including magneto-transport in low-dimensional materials, charge separation, and Hall effect-based devices. Introducing a central repulsive force proportional to the radial displacement significantly modifies this dynamic. The resulting potential for such a case is formally equivalent to an inverted 2D harmonic oscillator, namely, a harmonic oscillator with a negative spring constant [29]. Such a potential promotes instability and unbounded motion in the absence of confinement. When a perpendicular magnetic field is added, the resulting competition between magnetic field-induced rotation and repulsive acceleration provides a minimal yet versatile framework for describing non-equilibrium charge dynamics in nanostructured systems.

The interplay between central repulsion and magnetic confinement gives rise to a rich spectrum of dynamical regimes. While the magnetic field induces circular motion, it can also partially counteract the outward acceleration generated by the repulsive potential. As a result, the system exhibits a range of behaviors from rapid escape to quasi-confined orbits depending on the relative strength of the magnetic field and the repulsive interaction. Such coupled dynamics are relevant to nanoscale systems, including magnetically controlled charge transport, confined plasmas in micro- and nano-structures, and charged particle manipulation in low-dimensional devices [30,31,32,33]. From a theoretical perspective, the presence of the magnetic vector potential introduces a nontrivial coupling between position and canonical momentum, leading to complex phase-space structures that are important for understanding transport and stability in engineered nanomaterials.

In this work, we present an analytical and numerical investigation of the 2D motion of a charged particle subjected to a central repulsive potential and a perpendicular magnetic field. The present model should be viewed only as a classical framework for studying charged-particle motion under competing repulsive and magnetic effects in low-dimensional systems. The governing equations of motion are derived and solved, and the resulting trajectories are systematically classified as functions of system parameters. This combined force configuration provides a minimal yet versatile model for exploring non-equilibrium charge dynamics in low-dimensional environments. The results offer insights relevant to nanoscale transport, field-controlled carrier manipulation, and the design of nanostructured systems in which repulsive interactions and magnetic confinement can be engineered.

While the equations of motion are classical in nature, the novelty and physical insight of the present work lie in the specific interplay between a destabilizing inverted harmonic potential and magnetic field-induced confinement in a 2D geometry relevant to nanoscale transport environments. To our knowledge, this combined configuration has not been systematically analyzed in the context of charge transport and confinement in low-dimensional nanostructured systems. In contrast to standard cyclotron motion or conventional harmonic confinement problems, the competition between repulsive radial dynamics and Lorentz force-driven rotation generates a rich set of trajectory regimes, including spiraling escape, transient quasi-confinement, and parameter-sensitive transport behavior. Our analytical treatment provides explicit conditions governing these regimes and clarifies how magnetic fields can partially stabilize otherwise unstable motion. The new physical insight provided by this work is therefore not merely the solution of a classical dynamics problem, but the identification and classification of transport behaviors that may emerge in effective repulsive nanoscale environments, such as externally gated 2D systems, plasma-like nanostructures, or non-equilibrium charge-carrier configurations.

The article is organized as follows. In Section 2, we introduce the model and describe the solution method. In Section 3, we describe key results obtained for various parameter regimes. In Section 4, we summarize the work and present the main conclusions.

## 2. Model and Solution

We consider a charged particle of mass, m>0 and charge, q>0 moving in 2D space, under the influence of two forces. The first force is a central repulsive force of the form:

(1) $${\vec{F}}_{r}=k\;\vec{r},$$

where k>0 characterizes the strength of the repulsion and $\vec{r}=\left(x,y\right)$ is a 2D vector position. The charged particle is in a uniform magnetic field in the *z*-direction,

(2) $$\vec{B}=\left(0,0,B\right),$$

where $B\;=\;|\vec{B}|\;>0$ is the magnitude of the magnetic field. Such a magnetic field gives rise to the magnetic force:

(3) $${\vec{F}}_{B}=q\;\vec{v}\times \vec{B},$$

where $\vec{v}$ is the velocity of the particle. The central repulsive linear force considered in the present model can be physically interpreted as arising from an effective inverted harmonic oscillator potential that occurs in several realistic nanoscale and low-dimensional systems. In semiconductor nanostructures and 2D materials, externally applied gate voltages, charged impurities, local electrostatic engineering, or patterned electrode geometries can generate regions in which charge carriers experience an outward effective force. Near unstable equilibrium points, saddle-like electrostatic landscapes may also be approximated locally by an inverted harmonic potential, making the present model relevant as an idealized description of carrier dynamics in such environments. In addition, repulsive effective potentials may emerge in plasma-like nanosystems, non-equilibrium charge distributions, or driven mesoscopic structures where collective interactions produce locally unstable regions for charged-particle motion. Similar effective descriptions are also used in the study of anti-confinement configurations, magnetic focusing devices, and unstable transport channels in low-dimensional systems. While the present work is primarily theoretical, the inverted harmonic potential serves as a useful and physically motivated approximation for exploring how magnetic fields compete with destabilizing forces in nanoscale charge transport and confinement phenomena.

For this model, the initial 2D position and initial 2D velocity are, respectively, denoted as:

(4) $${\vec{r}}_{0}=\left(x_{0},y_{0}\right)$$

and

(5) $${\vec{v}}_{0}=\left(v_{0x},v_{0y}\right).$$

Combining both force contributions, the Newtonian equation of motion for the particle is:

(6) $$m\;\dot{\vec{v}}=q\;\vec{v}\times \vec{B}+k\;\vec{r},$$

where $\dot{\vec{v}}$ denotes the first derivative of velocity with respect to time. Writing this in component form:

(7) $$\left\{\begin{array}{c} m\;{\dot{v}}_{x}=q\;B\;v_{y}+k\;x\\ m\;{\dot{v}}_{y}=-q\;B\;v_{x}+k\;y. \end{array}\right.$$

These are two coupled second-order differential equations for x(t) and y(t). To simplify the notation, we can introduce natural scales. To this effect, let:

(8) $$\omega_{c}=\frac{qB}{m}\geq 0,$$

be the cyclotron frequency and

(9) $$\omega^{2}=\frac{k}{m}\geq 0,$$

be the repulsive force constant per unit mass. Then, the coupled differential equations become:

(10) $$\left\{\begin{array}{c} {\dot{v}}_{x}=+\omega_{c}\;v_{y}+\omega^{2}\;x\\ {\dot{v}}_{y}=-\omega_{c}\;v_{x}+\omega^{2}\;y. \end{array}\right.$$

At first sight, these equations describe a linearly unstable system (due to the repulsion), with rotation induced by the perpendicular magnetic field.

To solve the problem, in principle, one can always apply conventional differentiation methods, which in this case result in solving systems of linear differential equations [34]. While this is a worthy approach, we show in this work that the exact solution can be obtained more elegantly by using an alternative method that involves complex variables. We start the process by multiplying both sides of the second expression in Equation (10) by the imaginary number, $i=\sqrt{-1}$. The next step is to add side by side the two expressions in the same equation:

(11) $${\dot{v}}_{x}+i\;{\dot{v}}_{y}=-i\;\omega_{c}\;\left(v_{x}+i\;v_{y}\right)+\omega^{2}\;\left(x+i\;y\right).$$

Now, one defines a standard complex position variable:

(12) z=x+iy.

One can immediately see that the complex velocity is $v=v_{x}+i\;v_{y}=\dot{z}$ and complex acceleration is $\dot{v}={\dot{v}}_{x}+i\;{\dot{v}}_{y}=\ddot{z}$. Based on these results, one writes Equation (11) as

(13) $$\ddot{z}+i\;\omega_{c}\;\dot{z}-\omega^{2}\;z=0,$$

where the initial time conditions for the complex position and complex velocity are:

(14) $$z\left(t=0\right)=z_{0}=x_{0}+i\;y_{0}$$

and

(15) $$v\left(t=0\right)=v_{0}=v_{0x}+i\;v_{0y}.$$

The general solution of the above differential equation is given as:

(16) $$z\left(t\right)=c_{1}\;\exp\left(r_{1}\;t\right)+c_{2}\;\exp\left(r_{2}\;t\right),$$

where $r_{1}$ and $r_{2}$ are the roots of the following quadratic equation:

(17) $$r^{2}+i\;\omega_{c}\;r-\omega^{2}=0.$$

The two complex constants, $c_{1}$ and $c_{2}$ are determined from the initial conditions, $z\left(t=0\right)=z_{0}$ and $v\left(t=0\right)=v_{0}$. By solving for *r* in Equation (17), one has:

(18) $$r_{1,2}=-i\;\frac{\omega_{c}}{2}\pm \frac{1}{2}\;\sqrt{4\;\omega^{2}-\omega_{c}^{2}}.$$

For clarity, we remark that the first root, $r_{1}$, corresponds to the “+” sign. Consequently, the second root, $r_{2}$ corresponds to the “−” sign. It is clear that the nature of the solution depends on how ω compares to $\omega_{c}$. There are three possible regimes of motion, which we classify as the unbounded regime ($\omega >\frac{\omega_{c}}{2}$), quasi-confined regime ($\omega =\frac{\omega_{c}}{2}$) and bounded regime ($\omega <\frac{\omega_{c}}{2}$).

### 2.1. Unbounded Regime ($\omega >\frac{\omega_{c}}{2}$)

For such a case, one can write:

(19) $$r_{1,2}=-i\;\frac{\omega_{c}}{2}\pm \frac{\alpha }{2},$$

where

(20) $$\alpha =\sqrt{4\;\omega^{2}-\omega_{c}^{2}}>0,$$

is a positive real parameter. One may write the general solution as:

(21) $$z\left(t\right)=\exp\left(-i\;\frac{\omega_{c}}{2}\;t\right)\left[c_{1}\;\exp\left(\frac{\alpha }{2}\;t\right)+c_{2}\;\exp\left(-\frac{\alpha }{2}\;t\right)\right],$$

where the complex constants, $c_{1}$ and $c_{2}$, are determined from the initial time conditions for the complex position and complex velocity.

This scenario also includes the case of a vanishing magnetic field:

(22) $$\omega_{c}=0.$$

When $\omega_{c}=0$, the expression in Equation (21) takes the form:

(23) $$z\left(t\right)=c_{1}\;\exp\left(\omega \;t\right)+c_{2}\;\exp\left(-\omega \;t\right).$$

With the exception of the trivial case, $z_{0}=v_{0}=0$ which leads to $c_{1}=c_{2}=0$, the motion invariably ends at infinity.

### 2.2. Quasi-Confined Regime ($\omega =\frac{\omega_{c}}{2}$)

For such a case, the solution to the quadratic equation in Equation (17) leads to a double root:

(24) $$r_{1}=r_{2}=-i\;\frac{\omega_{c}}{2}.$$

This means that one solution of the starting differential equation can be written as:

(25) $$z_{1}\left(t\right)=\exp\left(-i\;\frac{\omega_{c}}{2}\;t\right).$$

By following standard techniques from differential equations [34], the second linearly independent solution is:

(26) $$z_{2}\left(t\right)=t\;z_{1}\left(t\right).$$

Thus, one may write the general solution as:

(27) $$z\left(t\right)=\exp\left(-i\;\frac{\omega_{c}}{2}\;t\right)\left(c_{1}+c_{2}\;t\right),$$

where the complex constants, $c_{1}$ and $c_{2}$, are determined from the initial time conditions for the complex position and complex velocity. The distance away from the center can be measured from the magnitude squared of the 2D complex position:

(28) $${\left|z\left(t\right)\right|}^{2}=z\left(t\right)\;z{\left(t\right)}^{*}={\left|c_{1}+c_{2}\;t\right|}^{2},$$

where the asterisk (*) means complex conjugation. We call this the quasi-confined regime because the escape to infinity is very slow, $|z\left(t\right)|\propto |\;c_{2}|\;t$ in the t→∞ limit. It is worth clarifying that the stated long-time behavior is the generic behavior for the corresponding parameter regime since special initial conditions may suppress some growing or linear terms.

### 2.3. Bounded Regime ($\omega <\frac{\omega_{c}}{2}$)

This case leads two different roots in Equation (18) that are both imaginary. We leave it to the reader to verify that one can write:

(29) $$r_{1,2}=-\frac{i}{2}\left(\omega_{c}\mp \Omega \right),$$

where

(30) $$\Omega =\sqrt{\omega_{c}^{2}-4\;\omega^{2}}>0,$$

is a positive real parameter. Note that:

(31) $$\omega_{c}\geq \Omega ,$$

with the equality occurring only when ω=0 given that $\omega_{c}>0$.

One may write the general solution as:

(32) $$z\left(t\right)=c_{1}\;\exp\left[-\frac{i}{2}\left(\omega_{c}-\Omega \right)t\right]+c_{2}\;\exp\left[-\frac{i}{2}\left(\omega_{c}+\Omega \right)t\right],$$

where the complex constants, $c_{1}$ and $c_{2}$, are determined from the initial time conditions for the complex position and complex velocity.

The case of cyclotron motion would correspond to:

(33) ω=0,

resulting in:

(34) $$z\left(t\right)=c_{1}+c_{2}\;\exp\left(-i\;\omega_{c}\;t\right).$$

A detailed pedagogical discussion of the cyclotron circular motion using complex notation was provided in a recent paper [28].

## 3. Results

In order to keep some degree of generality, we assume:

(35) $$\omega_{c}\neq 0.$$

For this choice, we can follow up on the evolution of motion from cyclotron circular motion to other regimes by varying the parameter ω starting from ω=0 (cyclotron motion) to $\omega \gg \omega_{c}$. In addition, many possible scenarios arise from an analysis of the equations of motion depending on the initial conditions. As a matter of fact, we can identify four possible initial starting conditions: (i) $z_{0}=0\;;\;v_{0}=0$; (ii) $z_{0}=0\;;\;v_{0}\neq 0$; (iii) $z_{0}\neq 0\;;\;v_{0}=0$; and (iv) $z_{0}\neq 0\;;\;v_{0}\neq 0$. These initial conditions vary from very simple as in (i) to the most general case as in (iv). For case (i), one can immediately say that if the particle is initially at rest ($v_{0}=0$) at the origin $\left(z_{0}=0\right)$, the particle will stay there forever, z(t)=0 and v(t)=0. At $z_{0}=0$, the force is zero. Newton’s second law gives an acceleration that is initially zero. So, if the particle is initially at rest at the origin, it will remain there forever. However, it is not easy at all to anticipate the resulting motion for all other cases (ii), (iii) and (iv) of initial conditions.

Thus, we direct our attention to a specific case and investigate the diverse possibilities of interest it entails. A general method to determine the arbitrary constants from the initial time conditions can be prescribed for the scenarios in Section 2.1 and Section 2.3. To start with, one writes the general solution as:

(36) $$z\left(t\right)=c_{1}\;f_{1}\left(t\right)+c_{2}\;f_{2}\left(t\right),$$

where $f_{1}\left(t\right)$ and $f_{2}\left(t\right)$ are the two linearly independent solutions, while $c_{1}$ and $c_{2}$ are two arbitrary complex constants to be determined from the initial starting conditions of z(t) and $v\left(t\right)=\dot{z}\left(t\right)$ at time t=0. For this particular setup, we have:

(37) $$z\left(t=0\right)=c_{1}\;f_{1}\left(t=0\right)+c_{2}\;f_{2}\left(t=0\right)=z_{0},$$

and

(38) $$v\left(t=0\right)=c_{1}\;{\dot{f}}_{1}\left(t=0\right)+c_{2}\;{\dot{f}}_{2}\left(t=0\right)=v_{0}.$$

One can express $c_{2}$ in terms of $c_{1}$ by applying the condition in Equation (37) for z(t) in Equation (36):

(39) $$c_{2}=\frac{z_{0}}{f_{2}\left(t=0\right)}-c_{1}\;\frac{f_{1}\left(t=0\right)}{f_{2}\left(t=0\right)}.$$

Note that this step is legitimate only when $f_{2}\left(t=0\right)\neq 0$. This means that this general method is not applicable for the case in Section 2.2 which must be treated as special. Returning to the scenario that applies to the cases in Section 2.1 and Section 2.3, one substitutes $c_{2}$ from Equation (39) into Equation (36) to obtain:

(40) $$z\left(t\right)=c_{1}\left[f_{1}\left(t\right)-\frac{f_{1}\left(t=0\right)}{f_{2}\left(t=0\right)}\;f_{2}\left(t\right)\right]+z_{0}\;\frac{f_{2}\left(t\right)}{f_{2}\left(t=0\right)}.$$

The constant, $c_{1}$, is determined from the initial condition for the velocity, $v\left(t\right)=\dot{z}\left(t\right)$ at time t=0. This way, one calculates that:

(41) $$c_{1}=\frac{v_{0}-z_{0}\;\frac{{\dot{f}}_{2}\left(t=0\right)}{f_{2}\left(t=0\right)}}{{\dot{f}}_{1}\left(t=0\right)-\frac{f_{1}\left(t=0\right)}{f_{2}\left(t=0\right)}\;{\dot{f}}_{2}\left(t=0\right)}.$$

This means that the solution in Equation (36) which satisfies the initial conditions in Equations (37) and (38) is given from Equation (40) with the complex constant, $c_{1}$ determined from Equation (41). One can finally obtain the positions of the particle, x(t) and y(t) as a function of time by separating the real and imaginary parts of the expression for the complex position, z(t) and taking into account that $e^{i\;\theta }=\cos\left(\theta \right)+i\;\sin\left(\theta \right)$.

For simplicity, we take

(42) $$\omega_{c}=1.$$

In order to explore all the possible trajectory regimes, we vary parameter, ω from values of ω=0 to ω≫1/2. Since there is a very large variety of possible initial conditions, we consider in detail only the case when motion starts from the origin and the initial velocity is along the *y*-direction. Thus, we consider the following initial condition written in complex notation as:

(43) $$z_{0}=0\;\;\;;\;\;v_{0}=i.$$

**Figure 1:**
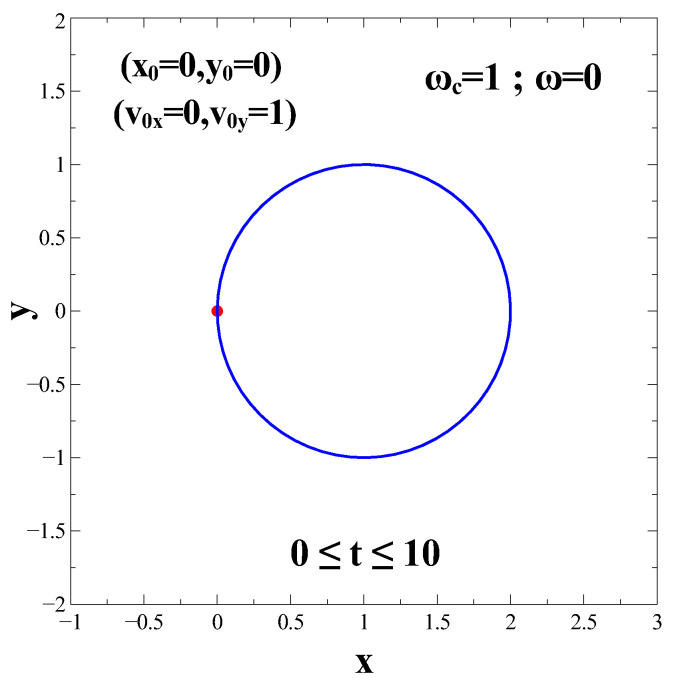
Trajectory of the particle for $\omega_{c}=1$, ω=0 and a time interval of 0≤t≤10. The initial position of the particle is at the origin, $\left(x_{0}=0,y_{0}=0\right)$ and is represented by a solid circle (red). The initial velocity is along the *y*-direction, $\left(v_{0x}=0,v_{0y}=1\right)$.

The case for ω=0 represents circular cyclotron motion as seen from Figure 1.

In all trajectory figures, the x- and y-axes represent the Cartesian coordinates of the charged particle in the 2D plane perpendicular to the applied magnetic field. More specifically, they describe the instantaneous position of the particle as it evolves under the combined action of the repulsive central linear force and the Lorentz force induced by the magnetic field.

**Figure 2:**
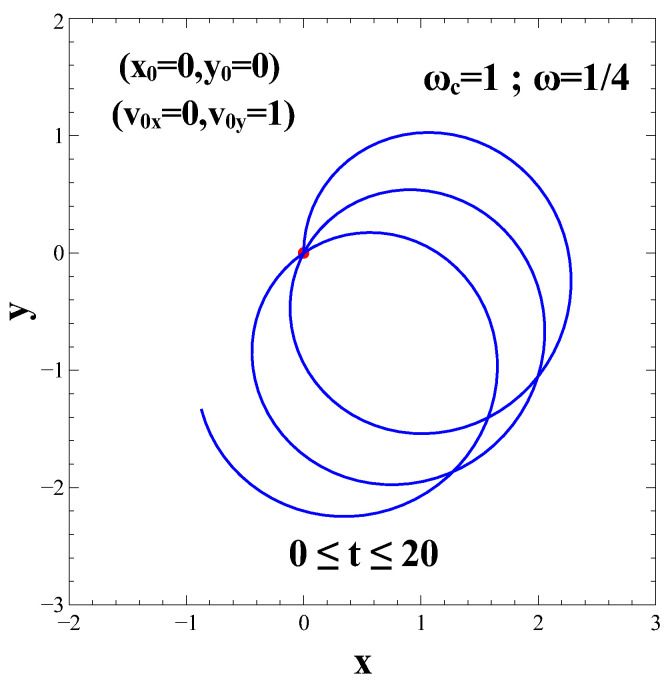
Trajectory of the particle for $\omega_{c}=1$, ω=1/4 and a time interval of 0≤t≤20. The initial position of the particle is at the origin, $\left(x_{0}=0,y_{0}=0\right)$ and is represented by a solid circle (red). The initial velocity is along the *y*-direction, $\left(v_{0x}=0,v_{0y}=1\right)$.

We now turn on the repelling force and consider a value of ω=1/4 (case $\omega <\omega_{c}/2$). The 2D trajectory of the charged particle for a time range, 0≤t≤20 is shown in Figure 2.

**Figure 3:**
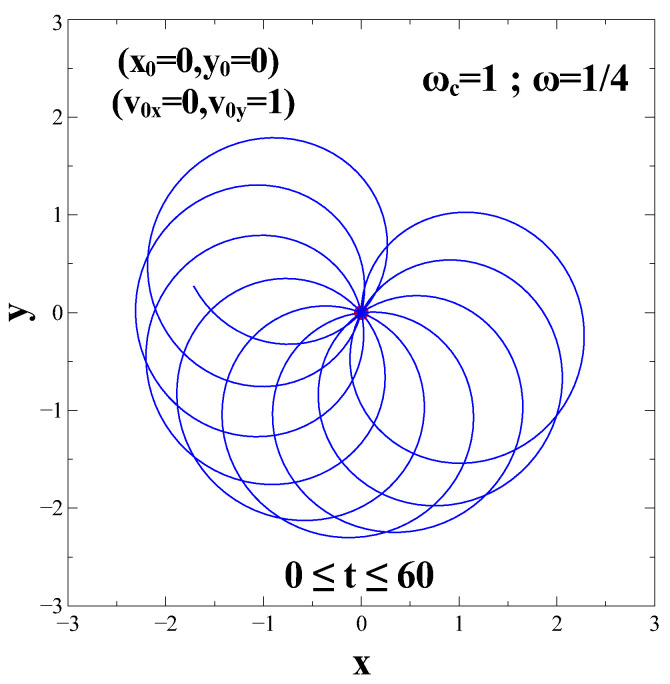
Trajectory of the particle for $\omega_{c}=1$, ω=1/4 and a time interval of 0≤t≤60. The initial position of the particle is at the origin, $\left(x_{0}=0,y_{0}=0\right)$ and is represented by a solid circle (red). The initial velocity is along the *y*-direction, $\left(v_{0x}=0,v_{0y}=1\right)$.

**Figure 4:**
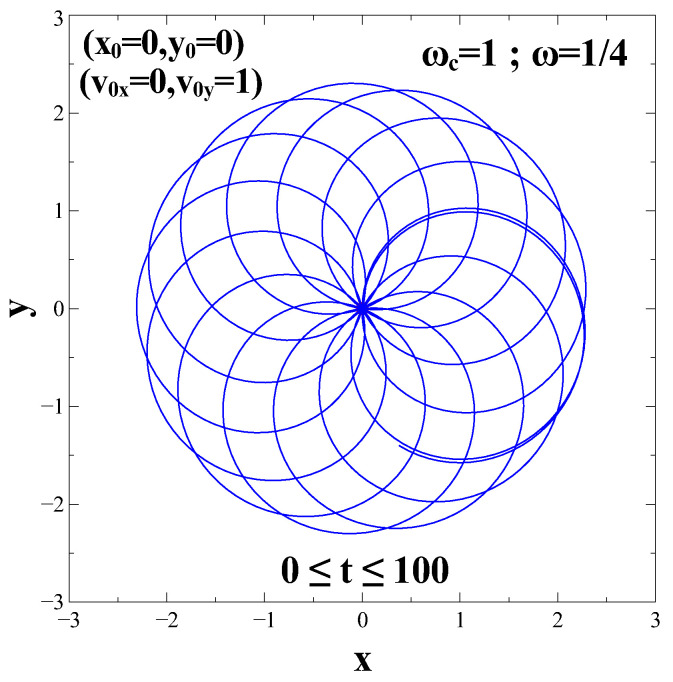
Trajectory of the particle for $\omega_{c}=1$, ω=1/4 and a time interval of 0≤t≤100. The initial position of the particle is at the origin, $\left(x_{0}=0,y_{0}=0\right)$ and is represented by a solid circle (red). The initial velocity is along the *y*-direction, $\left(v_{0x}=0,v_{0y}=1\right)$.

Note the time evolution of the 2D trajectory for a longer time range, 0≤t≤60 as shown in Figure 3. The result hints the existence of bound orbits as verified by looking at a longer time range, 0≤t≤100 as shown in Figure 4.

The appearance of the patterns exhibits similar features to those seen in earlier works where we considered a time-dependent magnetic field [35,36]. A time-dependent magnetic field induces an electric field and makes the problem more difficult.

**Figure 5:**
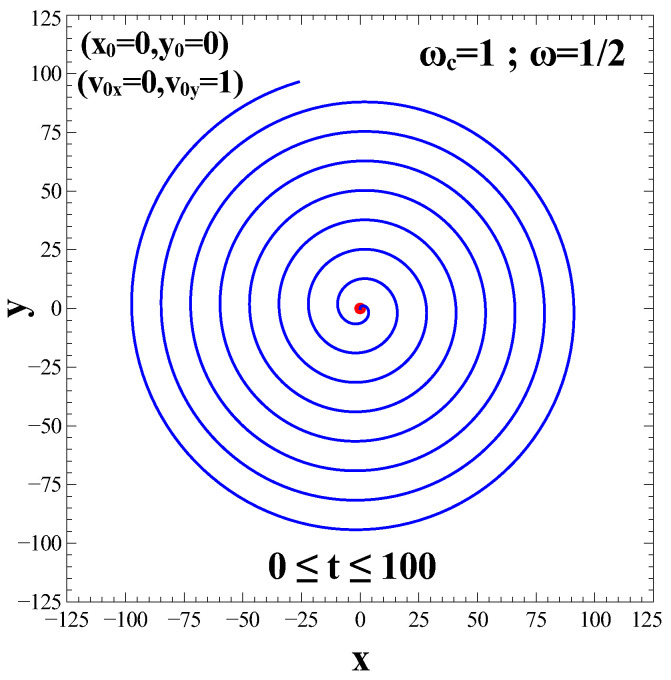
Trajectory of the particle for $\omega_{c}=1$, ω=1/2 and a time interval of 0≤t≤100. The initial position of the particle is at the origin, $\left(x_{0}=0,y_{0}=0\right)$ and is represented by a solid circle (red). The initial velocity is along the *y*-direction, $\left(v_{0x}=0,v_{0y}=1\right)$.

We now consider the very special value of ω=1/2 (case $\omega =\omega_{c}/2$). This represents the scenario in Section 2.2. A plot of the 2D trajectory of the charged particle for a time range, 0≤t≤100 is shown in Figure 5.

It can be proven that the resulting motion for such a case is a spiral of Archimedes.

**Figure 6:**
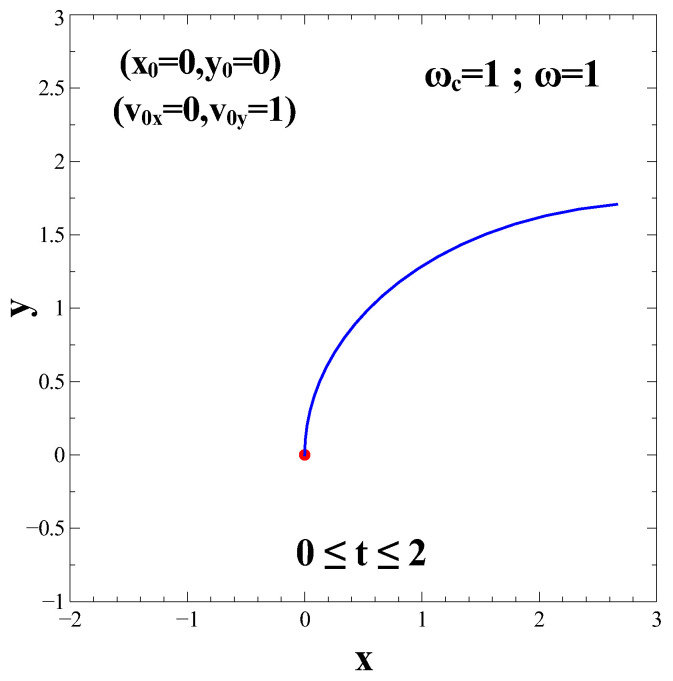
Trajectory of the particle for $\omega_{c}=1$, ω=1 and a time interval of 0≤t≤2. The initial position of the particle is at the origin, $\left(x_{0}=0,y_{0}=0\right)$ and is represented by a solid circle (red). The initial velocity is along the *y*-direction, $\left(v_{0x}=0,v_{0y}=1\right)$.

**Figure 7:**
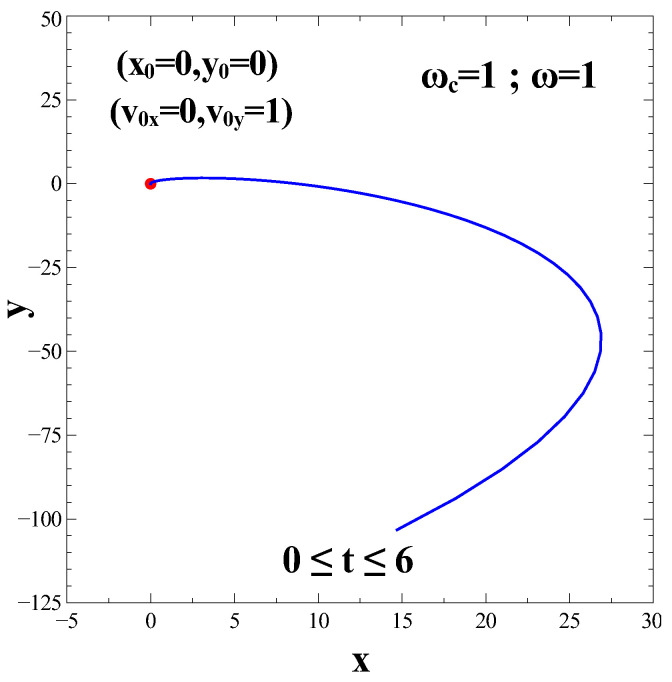
Trajectory of the particle for $\omega_{c}=1$, ω=1 and a time interval of 0≤t≤6. The initial position of the particle is at the origin, $\left(x_{0}=0,y_{0}=0\right)$ and is represented by a solid circle (red). The initial velocity is along the *y*-direction, $\left(v_{0x}=0,v_{0y}=1\right)$.

To finalize the analysis, let us also consider a value of ω=1 (case $\omega >\omega_{c}/2$). A plot of the 2D trajectory for a short time range, 0≤t≤2 is shown in Figure 6. A plot of the 2D trajectory for a longer time interval, 0≤t≤6 and at a much larger scale is shown in Figure 7. This plot indicates that the particle is pushed away from the center and the motion is not bound.

We expect that the effect of changing the initial position and initial velocity for various values will have a dramatic impact on the resulting 2D trajectories and the combinations are practically limitless. In particular, the 2D bound path patterns observed for $\omega <\omega_{c}/2$ are expected to be very elaborate, suggesting the possibility of many scenarios that depend on the interplay of ω, $\omega_{c}$ and the initial conditions, $z_{0}$ and $v_{0}$.

**Figure 8:**
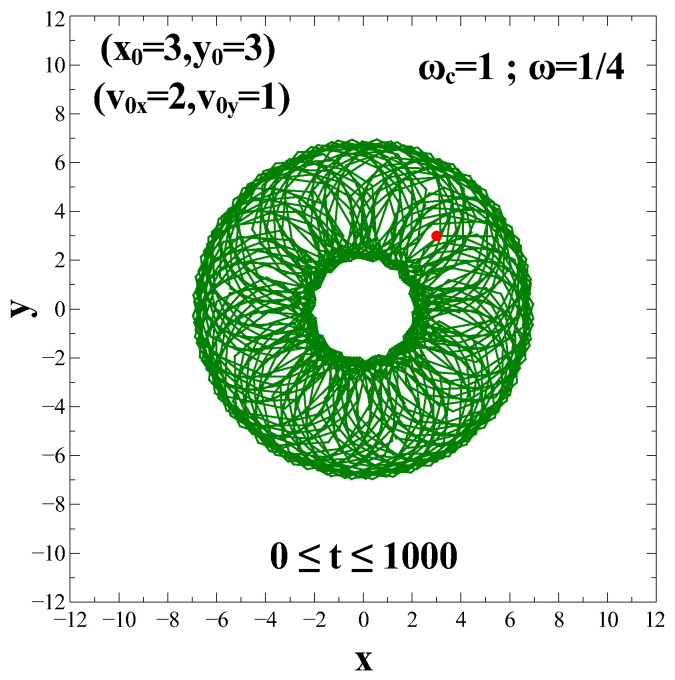
Trajectory of the particle for $\omega_{c}=1$, ω=1/4 and a time interval of 0≤t≤1000. The initial position of the particle is at $\left(x_{0}=3,y_{0}=3\right)$ and is represented by a solid circle (red). The initial velocity is not parallel to any of the axes, $\left(v_{0x}=2,v_{0y}=1\right)$. The data points are obtained in time intervals of Δt=1 for better visibility.

Let us now expand the analysis a little bit to include a broader range of initial positions and velocities in order to better demonstrate how the resulting trajectories depend on the full set of initial conditions. We want to explicitly illustrate how variations in the initial position, velocity magnitude, and velocity direction influence the trajectories. Changing the initial position and the initial velocity to more general values will have important effects, since there are many possible variations. As a good representative example, we consider values $\omega_{c}=1$ and ω=1/4 (case $\omega <\omega_{c}/2$). We take the initial position not at the origin, but away from it at $\left(x_{0}=3,y_{0}=3\right)$. Similarly, we consider the initial velocity not to be parallel to any of the axes, $\left(v_{0x}=2,v_{0y}=1\right)$. A plot of the 2D trajectory for a long time range, 0≤t≤1000 is shown in Figure 8. One notes that motion, as expected, is bound. However, the more general initial conditions lead to different patterns. The appearance of a central “forbidden” region is most noticeable for this particular case. While initial conditions impact the form of the trajectories, we want to clarify that the analytical solutions derived in this work are fully general and valid for arbitrary initial conditions. The trajectory classifications (for instance, bound or unbound) are fundamentally determined by the system parameters, such as magnetic-field strength and repulsive potential amplitude. However, the form of the resulting trajectories is heavily influenced by the relative contribution of the independent solution modes fixed by the chosen initial state. Obviously, the case with more general initial conditions is useful only as an illustrative example and does not constitute a complete survey of all possible initial states.

At this juncture, it is important to remark that trajectory plots are useful for illustration purposes but they do not fully characterize the dynamics of the system. The three regimes already identified are the ones that provide a quantitative classification of the motion where one can explicitly distinguish unbounded ($\omega >\omega_{c}/2$), quasi-confined ($\omega =\omega_{c}/2$) and bounded regimes ($\omega <\omega_{c}/2$) based on the analytical structure of the frequencies obtained from the coupled equations of motion. In particular, we can rigorously clarify the parameter conditions under which the effective frequencies remain purely oscillatory or acquire exponentially growing components, thereby determining the transition between stable and unstable dynamics. The dependence of various quantities on the magnetic-field strength and repulsive force parameters, namely, the relative value of ω with respect to $\omega_{c}/2$ allows the different dynamical regimes to be classified quantitatively rather than only visually.

## 4. Conclusions

We investigate the classical 2D dynamics of a charged particle subjected to a central repulsive force proportional to the radial distance and a uniform magnetic field applied perpendicular to the plane. The particle motion remains confined to the 2D plane, where the competition between the destabilizing repulsive interaction and the Lorentz force-induced rotation governs the trajectory. The resulting equations of motion form a coupled set of differential equations that are nontrivial to solve using standard approaches. To address this, we employ a complex-variable formalism in which the 2D position and velocity are represented as complex quantities. This method reduces the problem to a single linear second-order differential equation with constant complex coefficients for the complex position, enabling an exact and compact analytical treatment. The obtained solutions exhibit a range of trajectory types, including unbounded motion, spiral paths, and parameter-dependent quasi-confined orbits.

The system can be interpreted as a charged-particle analogue of an anti-harmonic oscillator, where intrinsic instability is partially mitigated by magnetic confinement. Depending on the relative strength of the repulsive interaction and magnetic field, three distinct dynamical regimes are identified and analyzed. The results highlight the sensitivity of trajectories to initial conditions and system parameters, revealing complex phase-space behavior. This framework provides a useful model for studying non-equilibrium charge dynamics in low-dimensional systems, with potential relevance to nanoscale transport, plasma-like confinement in nanostructures, and field-controlled manipulation of charge carriers. It also offers insight into how externally applied magnetic fields can regulate or transform intrinsically unstable dynamics in engineered nanomaterials.

At this stage, we take the opportunity to reiterate the main novelty of the present work, which lies in the analytical investigation of charged-particle dynamics in a 2D system subject to a combination of an inverted harmonic (repulsive) potential and a perpendicular magnetic field. This configuration has received comparatively little attention, despite its clear relevance to nanoscale transport and confinement phenomena. Unlike standard studies focusing solely on cyclotron motion or harmonic confinement, our model explores the competition between destabilizing radial repulsion and magnetic field-induced rotational confinement, giving rise to rich dynamical regimes such as spiraling escape, transient quasi-confined motion, and parameter-dependent transport behaviors. A further novel aspect of this work is the complete analytical classification of the resulting trajectories in terms of system parameters. We identify the precise conditions under which magnetic effects can partially stabilize motion that would otherwise be unstable and provide explicit expressions for the characteristic frequencies and growth behavior of the trajectories. This study identifies the conditions under which magnetic effects can partially stabilize otherwise unstable motion and provides explicit expressions for the characteristic frequencies and growth behavior of the trajectories. In this way, the work offers a physically transparent framework for understanding charge-carrier motion in low-dimensional nanostructured environments where effective repulsive interactions may emerge from external gating, collective effects, or non-equilibrium conditions.

## Data Availability

The data are available upon request at ogciftja@pvamu.edu.
